# Cremation or Burial?

**Published:** 1897-06

**Authors:** 


An animated discussion as to whether cremation or burial should be adopted in the disposal of the dead has been recently indulged by one of our dailies. One writer claims that burial and sepulture are not antagonistic to sanitation, and that cremation defiles the air. An argument strongly in favor of the burial of the dead lies in the well-known fact that the health of Paris has never suffered by reason of its catacombs. The city of Brooklyn, possesssing numerous burial grounds, is one of the healthiest communities that one can live in. On the other hand, the gaseous fumes of a London crematory have been known to be prejudicial to the health of those living near by. This problem offers many points of interest, both to the layman and the scientist.

